# Personalized Hemodialysis Approaches in Frail Older Individuals

**DOI:** 10.3390/geriatrics11020040

**Published:** 2026-04-07

**Authors:** Guido Gembillo, Luca Soraci, Matteo Floris, Lorenzo Lo Cicero, Claudia Lo Re, Elvira Filicetti, Michela Calderone, Carmelo Giorgio Benenati, Andrea Corsonello, Domenico Santoro

**Affiliations:** 1 Unit of Nephrology and Dialysis, Department of Clinical and Experimental Medicine, University of Messina, 98125 Messina, Italy; lorenzolocicero95@gmail.com (L.L.C.); loreclaudia96@gmail.com (C.L.R.); mic.cald13@gmail.com (M.C.); giorgio.benenati@libero.it (C.G.B.); dsantoro@unime.it (D.S.); 2 Unit of Geriatric Medicine, Italian National Research Center on Aging (IRCCS INRCA), 87100 Cosenza, Italy; l.soraci@inrca.it (L.S.); e.filicetti@inrca.it (E.F.); a.corsonello@inrca.it (A.C.); 3 Department of Nephrology, Dialysis, and Transplantation, ARNAS G. Brotzu, 09134 Cagliari, Italy; matteo.floris@unica.it; 4 Department of Pharmacy and Health and Nutritional Sciences, University of Calabria, 87036 Rende, Italy

**Keywords:** frailty, older adults, hemodialysis, hemodiafiltration, ultrafiltration, personalized dialysis, vascular access, conservative kidney management, elderly, geriatric

## Abstract

The hemodialysis population has progressively aged over the past two decades; in several settings, adults aged ≥75 years represent one of the fastest-growing populations receiving dialysis. Frailty, characterized by reduced physiological reserve and heightened vulnerability to stressors, has emerged as a critical determinant of outcomes and is commonly assessed using validated instruments such as the Fried Frailty Phenotype or the Clinical Frailty Scale (CFS). Reported frailty prevalence in hemodialysis varies widely (approximately 20% to >80%), largely depending on the assessment instrument and the population studied, with consistently higher prevalence in older cohorts. It is consistently associated with older age, female sex, diabetes, lower serum albumin, cardiovascular disease, longer dialysis vintage, and lower physical activity. Compared with non-frail patients, frail hemodialysis patients have a substantially higher risk of death (approximately two-fold in pooled analyses). Seminal trials and large observational programs that shaped hemodialysis targets underrepresented very old, frail, and highly comorbid patients, limiting generalizability. In frail older adults with limited life expectancy and substantial comorbidity burden, standard thrice-weekly schedules, higher ultrafiltration intensity, and a uniform ‘fistula-first’ approach may increase treatment burden without clear proportional gains in patient-centered outcomes. This review examines evidence supporting individualized hemodialysis strategies in frail older adults. As the dialysis population continues to age, proficiency in goal-concordant, personalized prescribing is increasingly important for nephrologists and dialysis teams.

## 1. Introduction

### 1.1. The Aging Dialysis Population

The demographic landscape of end-stage kidney disease has shifted dramatically. The proportion of patients initiating or receiving dialysis at older ages has risen substantially worldwide [1]. According to the US Renal Data System, the adjusted prevalence of end-stage renal disease reached 2242 cases per million in 2018, with older individuals comprising a growing share of this population [1,2]. This trend reflects both better survival rates among dialysis patients and increased chronic kidney disease incidence in older adults with diabetes, hypertension, and cardiovascular comorbidities.

Frailty has become a defining characteristic of this population, though reported prevalence varies widely by setting and assessment tool. Chan et al.’s review in Kidney International documents estimates ranging from approximately 20% to over 80% across different cohorts and instruments [3,4,5]. A meta-analysis by Bossola et al. found that frailty affects 41% of older adults on maintenance hemodialysis globally [6]. This contrasts sharply with the 10.7% prevalence seen in community-dwelling older adults [7], indicating that hemodialysis patients face a 2–6-fold higher frailty risk.

### 1.2. The Mismatch Between Evidence and Practice

The seminal trials and large observational programs that established adequacy standards and practice targets, including the HEMO Study and DOPPS (Dialysis Outcomes and Practice Patterns Study), enrolled relatively few very old, frail, or highly comorbid patients, limiting their generalizability to this population [8]. While these investigations defined benchmarks for dialysis adequacy, session duration, and ultrafiltration targets, such standards may not apply to frail older patients who experience heightened sensitivity to hemodynamic shifts, prolonged recovery times, and increased access complications. For these individuals, treatment goals increasingly prioritize quality of life over longevity.

Recent evidence challenges the assumption that intensive dialysis universally benefits older patients. Studies show inconsistent improvements in patient-reported outcomes with increased dialysis intensity, and biochemical gains may not translate to better function or reduced symptom burden in frail individuals [5]. For older patients, health-related quality of life domains take precedence: alleviating symptom burden, preserving temporal autonomy, and maintaining functional independence often supersede survival maximization as primary treatment objectives [9]. This disconnect between protocol-driven metrics and patient values demands a shift toward personalized care.

However, personalization should not be interpreted as systematic de-intensification of dialysis therapy. Inappropriately reducing treatment intensity may expose frail patients to the risk of undertreatment or therapeutic nihilism. Rather, the goal of individualized care is to align dialysis intensity with clinical tolerance, prognosis, and patient priorities while maintaining adequate metabolic and volume control.

This review synthesizes evidence supporting individualized hemodialysis prescription in frail older adults. We begin by addressing frailty assessment, examining validated tools and their integration into nephrology practice. We then explore domains where personalization proves most critical: dialysis frequency and session duration, ultrafiltration strategies, vascular access selection, and the option of conservative kidney management. Throughout, we emphasize shared decision-making that incorporates patient preferences, functional status, and realistic prognostic expectations alongside traditional clinical metrics.

Although several observational studies and subgroup analyses have explored dialysis outcomes in older populations, robust randomized controlled trials specifically designed for frail patients remain scarce. As a consequence, many clinical decisions in this population rely on extrapolation from studies conducted in younger and less complex cohorts. This article presents a narrative review of the current literature on personalized hemodialysis strategies in frail older adults, aiming to summarize emerging evidence and clinical perspectives rather than to provide systematic evidence synthesis.

## 2. Frailty Assessment in Hemodialysis Patients

### 2.1. Defining Frailty

Frailty is a clinical syndrome characterized by a decline in physiological and cognitive reserve, manifesting as weight loss, exhaustion, low physical activity, weakness, and slow walking speed [5]. Unlike disability, defined as the inability to perform activities of daily living or comorbidity, the presence of multiple concurrent diseases, or frailty captures vulnerability to stressors and diminished capacity for recovery. Among hemodialysis patients, frailty confers not only reduced quality of life but also substantially elevated risks of hospitalization, infection, cardiovascular events, dialysis-related complications, and death [10,11].

### 2.2. Assessment Tools

Several instruments have been validated for frailty assessment in hemodialysis populations; each has distinct advantages (Table 1).

A comprehensive comparison study found that while different frailty scales yield varying prevalence estimates, most correlate with increased mortality, hospitalization, and cardiovascular events [16]. The choice of tool should balance practical constraints, clinical context, and desired precision.

### 2.3. Prognostic Value

Frailty is an independent predictor of poor outcomes in hemodialysis patients. Li et al. followed older hemodialysis patients prospectively for 12 months and found that frailty was associated with all-cause mortality, emergency department visits, and hospitalizations [17]. Multiple studies have reported hazard ratios for mortality in frail versus non-frail patients between 1.66 and 2.83 [6,18], and these associations persisted after adjusting for age and comorbidities. This suggests frailty adds prognostic information beyond traditional risk factors.

The effects of frailty go beyond mortality. Frail patients have more intradialytic complications and take longer to recover after dialysis sessions [19,20]. Symptom burden is higher and functional status declines rapidly in these patients. Recent work has also linked frailty to cognitive problems, depression, and poor sleep in the hemodialysis population [21].

### 2.4. Implementation in Clinical Practice

Despite its prognostic importance, systematic frailty assessment remains underutilized in nephrology practice. Barriers include time constraints, lack of training, and uncertainty about how to act on results [22]. Simple approaches exist for integration though. The CFS can be scored quickly during routine clinic visits, and the FRAIL scale takes less than 5 min to complete. Using these tools in pre-dialysis evaluation and periodic reassessment enables risk stratification that informs treatment planning.

**Figure 1:**
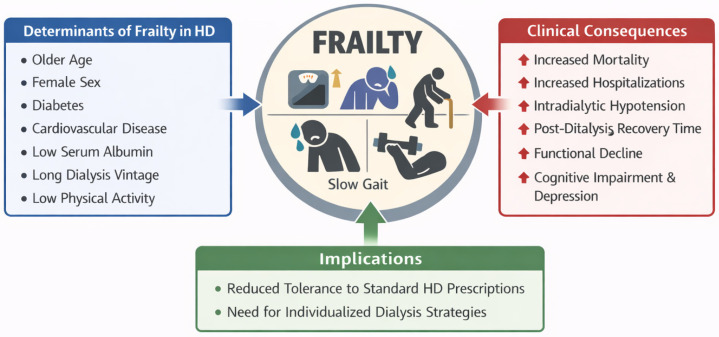
Frailty as a central modifier of outcomes in older hemodialysis patients.

When frailty is identified, the assessment should trigger several actions. These include comprehensive geriatric evaluation with medication review, fall risk assessment, and cognitive screening. Treatment goals and preferences need to be discussed through shared decision-making. Modifications to the dialysis prescription should be considered, along with the involvement of multidisciplinary team members such as social workers, physical therapists, and palliative care specialists when appropriate (Figure 1).

Figure 1 Conceptual framework illustrating frailty as a central vulnerability state in older hemodialysis patients. Determinants of frailty (left) converge on a frailty phenotype characterized by reduced physiological reserve, which is associated with adverse clinical consequences (right), including increased mortality, hospitalization, intradialytic instability, prolonged recovery time, functional decline, and cognitive impairment. Identification of frailty highlights reduced tolerance to standard hemodialysis prescriptions and supports the need for individualized treatment strategies.

## 3. Dialysis Prescription: Modality, Frequency and Session Duration

Selecting the most appropriate extracorporeal modality is a fundamental first step in dialysis prescription for frail older individuals, as different techniques offer distinct profiles of hemodynamic stability, symptom tolerance, and middle-molecule clearance.

High-flux hemodialysis remains widely used because of its availability, technical simplicity, and adequate clearance of small and middle molecules; however, compared with convective therapies, it has been associated with a higher incidence of intradialytic hypotension, muscle cramps, and treatment intolerance in older adults [23,24].

Hemodiafiltration (HDF), by integrating diffusive and convective clearance, has demonstrated improved intradialytic tolerance in older individuals, with fewer episodes of symptomatic hypotension, better middle-molecule removal (including β2-microglobulin), and enhanced dialysis efficiency when high convection volumes are achieved [25]. Recent randomized evidence further suggests that, compared with high-flux hemodialysis and when delivered at high convection volumes (≥23 L/session) HDF may reduce all-cause, cardiovascular, and infection-related mortality, with particularly consistent benefits in older patients (≥65 years), non-diabetic individuals, and those without established cardiovascular disease [23,26,27]. These effects may relate to superior removal of middle molecules and protein-bound uremic toxins, attenuation of chronic inflammation, and potential preservation of immune competence. Nevertheless, benefits appear volume-dependent and may not extend to all subgroups, reinforcing the need for careful patient selection [28].

Expanded dialysis using medium-cut-off membranes (HDx) and acetate-free biofiltration (AFB) has emerged as a potentially valuable option for older and frail hemodialysis patients [29]. Available evidence suggests that these techniques may improve intradialytic tolerability compared with conventional high-flux bicarbonate hemodialysis, with fewer symptomatic hypotensive episodes and better hemodynamic stability [30,31,32]. AFB also appears to provide more effective correction of metabolic acidosis. Data on survival are still limited; however, registry analyses have suggested an association between AFB and improved outcomes in older cohorts, although confirmatory randomized trials are lacking [33].

Compared with HDF, HDx and AFB may offer practical advantages in frail older individuals. HDx enhances middle-molecule removal through intrinsic membrane properties, without requiring high convection volumes, substitution fluid, or elevated blood flow rates [29,34]. This may be relevant in patients with limited vascular access performance or reduced cardiovascular reserve, in whom achieving the convection targets necessary to maximize HDF benefit is challenging. AFB combines effective solute removal with acetate-free bicarbonate delivery and has been associated with improved intradialytic stability, fewer intolerance symptoms, and superior correction of metabolic acidosis compared with standard bicarbonate dialysis and, in some series, HDF [35].

Thrice-weekly hemodialysis for 3.5–4 h per session has become the most widely used prescription, rooted in observations linking longer, more frequent dialysis with improved solute clearance and biochemical control. This standard imposes substantial burden though: twelve hours weekly in the dialysis unit, plus travel time, vascular access punctures, hemodynamic stress, and post-dialysis fatigue requiring hours of recovery [36]. For frail older individuals, these burdens may outweigh benefits. Autonomic dysfunction increases risk of intradialytic hypotension, while decreased cardiovascular reserve limits compensation for volume shifts [37]. Muscle wasting and malnutrition reduce interdialytic weight gain and preserved residual kidney function often reduces ultrafiltration needs. Post-dialysis recovery time affects 47–73% of patients in some cohorts and substantially impairs quality of life and functional independence [38].

Incremental hemodialysis challenges this one-size-fits-all approach by tailoring treatment frequency to individual needs, particularly residual kidney function. This typically begins with twice-weekly sessions, with transition to thrice-weekly occurring when residual function declines or uremic symptoms develop. At the other end of the spectrum, some patients benefit from longer, slower dialysis sessions such as nocturnal hemodialysis or more frequent short sessions. Increased frequency requires durable vascular access and adequate patient or caregiver capacity, which may limit feasibility in frail individuals. For motivated patients with adequate support, however, frequent short sessions may offer advantages over conventional schedules.

Home hemodialysis may represent an additional option for carefully selected older patients with adequate functional capacity and caregiver support. However, its feasibility in frail populations may be limited by functional impairment, caregiver burden, and logistical constraints.

A personalized approach is needed to optimize outcomes and improve tolerability in frail older individuals receiving hemodialysis (Table 2).

**Figure 2:**
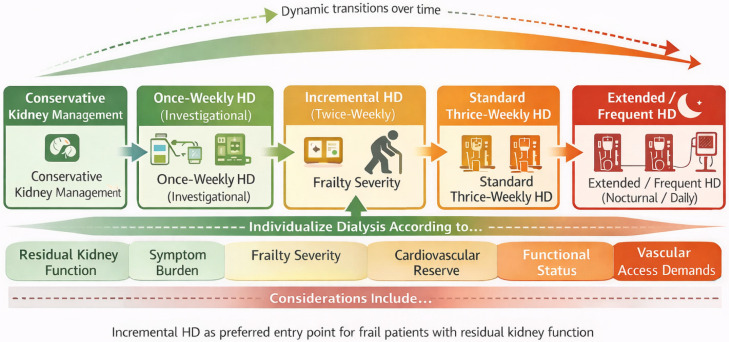
Spectrum of hemodialysis intensity and treatment burden in frail older patients.

The key principle is matching treatment intensity to individual circumstances. For frail patients with residual kidney function, minimal uremic symptoms, and difficulty tolerating standard schedules, incremental hemodialysis offers a rational starting point. Those with anuric kidney disease may still require standard thrice-weekly sessions, but session duration and ultrafiltration rates can be modified based on tolerance. The critical step is abandoning rigid adherence to population-level guidelines in favor of individualized assessment that considers residual renal function, interdialytic weight gain, functional status, symptom burden, and patient preferences (Figure 2).

Figure 2 Schematic representation of the spectrum of kidney replacement and supportive care strategies in frail older patients, ordered by increasing dialysis intensity and treatment burden. Approaches range from conservative kidney management and investigational low-frequency hemodialysis to incremental, standard, and extended or frequent hemodialysis. Dialysis intensity is individualized based on residual kidney function, frailty severity, symptom burden, cardiovascular reserve, functional status, and patient preferences, with dynamic transitions possible over time.

## 4. Ultrafiltration Strategies

### 4.1. The Problem with Rapid Fluid Removal

High ultrafiltration rates (UFR) during hemodialysis are a double-edged sword. They are necessary for volume management but associated with substantial morbidity and mortality. Landmark studies established that UFR > 13 mL/kg/hour correlates with increased risk of death [45,46]. The mechanism involves excessive intravascular volume depletion. When ultrafiltration velocities exceed the rate of plasma refilling from interstitial fluid compartments, the result is intravascular hypovolemia that manifests clinically as intradialytic hypotension, myocardial stunning, cerebral hypoperfusion, and accelerated decline in residual kidney function from repetitive ischemic insults [47,48].

Older frail patients are particularly vulnerable. Yu et al. conducted a 5-year cohort study showing that high UFR-induced intradialytic hypotension predicts cardiac remodeling, and older individuals had lower UFR thresholds for adverse events [49]. Age-related autonomic dysfunction compromises compensatory mechanisms, along with reduced cardiac reserve and structural heart disease [50].

The incidence of intradialytic hypotension ranges from 10% to 70% of hemodialysis sessions depending on definition [51]. A prospective study found 39.9% of maintenance hemodialysis patients experienced frequent hypotensive episodes, with older age and higher UFR among the independent risk factors [52]. The consequences extend beyond immediate symptoms to include longer recovery times, reduced quality of life, and increased all-cause mortality [53,54].

### 4.2. Weight-Specific UFR Thresholds

Recent analyses reveal that UFR-associated mortality risk varies by patient weight and sex, which challenges simple threshold recommendations. A cohort of 396,358 hemodialysis patients showed that mortality risk associated with ultrafiltration rate is not uniform across body weights. Mermelstein et al. established that the ultrafiltration rate corresponding to a 20% mortality hazard ratio follows the formula 3 W + 500 mL/hour (W = postdialysis weight in kg), with women having thresholds 70 mL/hour lower than men [55]. Heavier older patients also showed lower risk-associated UFR thresholds.

These findings highlight the limitations of universal UFR targets and support individualized approaches. The 13 mL/kg/hour benchmark is useful as a warning signal, but it should not drive clinical decisions without considering patient-specific factors like cardiovascular function, body composition, and hemodynamic tolerance [56].

### 4.3. Strategies to Reduce UFR and Prevent Hypotension

Multiple strategies can mitigate UFR-related complications. Extending dialysis sessions from 3.5 to 4.5–5 h proportionally reduces UFR and improves hemodynamic stability. This approach is supported by DOPPS findings linking longer treatment time to reduced mortality [57]. Although scheduling constraints and patient preferences may limit feasibility, it remains an important option for patients with recurrent hypotension.

Increasing treatment frequency to four or more sessions per week is another strategy that spreads fluid removal across additional treatments and lowers per-session UFR. This approach is particularly useful for patients with large interdialytic weight gains who cannot extend the duration of individual sessions.

Accurate assessment of dry weight is essential. Clinical parameters, including blood pressure patterns, edema, dyspnea, and post-dialysis fatigue, should be integrated with objective tools such as bioimpedance analysis [58]. Regular reassessment prevents both underestimation, which leads to chronic volume overload, and overestimation, which drives excessive UFR in pursuit of unattainable targets.

Dietary measures are also critical. Education about sodium restriction and fluid intake reduces interdialytic weight gain and helps achieve safer UFR targets. In patients with residual kidney function, loop diuretics may further support volume control.

Dialysate modifications can also help. Cooling dialysate from 37 °C to approximately 35.5 °C reduces intradialytic hypotension by limiting vasodilation [59]. Some centers use sodium or bicarbonate profiling to modulate intradialytic osmotic shifts, although supporting evidence remains mixed [60].

Finally, blood volume monitoring systems can support individualized ultrafiltration management. By continuously assessing relative blood volume, these systems, often integrated with biofeedback algorithms, enable dynamic adjustments to ultrafiltration and dialysate parameters to maintain hemodynamic stability and reduce the risk of intradialytic hypotension, particularly in hypotension-prone patients [61]. Randomized studies and meta-analyses suggest a reduction in IDH in selected populations, although benefits are less clear in fluid-overloaded or hypertensive patients. Multimodal assessment remains essential, as RBV monitoring does not fully distinguish between intravascular and interstitial compartments [62].

### 4.4. Accepting Higher Dry Weights in Frail Patients

Perhaps one of the most important strategies involves reconsidering dry weight targets. Aggressive dry weight goals may be inappropriate for frail older individuals. Traditional nephrology emphasizes euvolemia but pursuing this through rapid ultrafiltration that causes recurrent hypotension, myocardial stunning, and prolonged recovery seems counterproductive. Patients prioritize functional capacity and quality of life [63]. In selected frail individuals, modest relaxation of dry-weight targets may be preferable to repeated symptomatic hypotension and prolonged post-dialysis recovery. However, this strategy requires careful clinical monitoring, as persistent volume overload may contribute to hypertension, left ventricular remodeling, and heart failure progression. Achieving an appropriate balance between hemodynamic tolerance and long-term cardiovascular risk therefore remains a central challenge in dialysis prescription for frail patients. This does not mean neglecting volume management. It means balancing biochemical targets against clinical outcomes that matter to patients (Table 3).

## 5. Vascular Access Decisions

### 5.1. The Fistula-First Paradigm

The Fistula-First Initiative strongly prioritized arteriovenous fistulas (AVF) over arteriovenous grafts (AVG) and central venous catheters (CVC) for all hemodialysis patients [66]. This recommendation is supported by compelling evidence. AVFs have superior long-term patency, lower infection rates, and fewer need for interventions than other access types [67,68]. Mortality is also lower. These advantages reduce healthcare costs and improve patient outcomes, but only if the AVF matures successfully and provides long-term function.

The fistula-first approach faces important challenges in older populations. AVF maturation requires 2–6 months, and patients remain catheter-dependent during this period. Primary failure rates, defined as the inability to achieve usable maturation, reach 20–60% across studies. Older individuals fare even worse [69,70]. A study of octogenarians reported radiocephalic AVF primary failure rates of 42.1% versus 5.6% for brachiocephalic fistulas [71]. After successful maturation, AVFs still require frequent interventions to maintain patency, especially in older individuals with vascular disease.

### 5.2. Reconsidering Access Choices in Frail Older Individuals

Recent evidence challenges universal fistula-first application in older populations. Frail patients with limited life expectancy may not derive sufficient benefit to justify prolonged catheter dependence during AVF maturation, especially given high failure rates.

Lee et al. compared outcomes in 9458 older individuals (≥67 years) who initiated hemodialysis with a catheter and later received either AVF or AVG placement [72]. During the first 6 months, when catheter use was highest while awaiting maturation, AVF patients had higher cumulative catheter dependence than AVG patients. AVF patients ultimately had lower infection-related hospitalization rates and reduced mortality, but they were initially catheter-dependent for longer.

Lyu et al. looked at catheter dependence patterns in older individuals over 18 months of follow-up [73]. AVF placement was associated with significantly greater catheter dependence than AVG placement. This prolonged catheter exposure poses serious risks including catheter-related bloodstream infections, bacteremia, endocarditis, and higher mortality. Patient characteristics, such as life expectancy should inform access decisions rather than applying a one-size-fits-all approach.

### 5.3. The Role of Frailty Assessment

Frailty is an important consideration in vascular access selection. McDonnell et al. looked at frailty screening for hemodialysis access placement and found that frail patients had a higher mortality risk within 3 years of access creation [74]. Using the Risk Assessment Index (RAI) frailty score, they showed that frail and very frail patients had higher AVF non-maturation rates and shorter survival. AVG may be better for these patients.

The survival advantage of AVF over AVG requires sufficient life expectancy to offset the longer maturation time and higher primary failure rates [75]. For frail patients with multiple comorbidities and limited life expectancy, roughly 12–18 months or less, AVG or even definitive catheter use may make more sense. Subjecting these patients to procedures unlikely to benefit them over their remaining lifespan seems difficult to justify.

### 5.4. Toward Shared Decision-Making

The 2019 KDOQI guideline update moved away from rigid fistula-first toward a life-plan approach based on shared decision-making [76]. This framework considers patient life expectancy, functional status, vascular anatomy, and individual preferences.

Patients with a life expectancy of under 1 year derive limited benefit from AVF creation. Very frail patients face higher procedural risk and higher non-maturation rates, which reduces the opportunity to realize long-term AVF advantages. Poor vascular anatomy predicts AVF failure independent of other patient characteristics.

Individual preferences must be considered in access planning. Some patients prioritize avoiding repeated procedures or needle cannulation, while others prioritize minimizing catheter duration. Patients requiring urgent dialysis initiation often need temporary catheter placement with subsequent access reassessment once clinical stability is achieved.

### 5.5. Practical Recommendations

For frail older individuals starting hemodialysis, a reasonable approach begins with a comprehensive assessment of life expectancy, frailty status, vascular anatomy, and functional capacity. Patients need realistic counseling about what AVF creation involves. Maturation typically takes 3–6 months. Failure rates for distal AVF in older individuals can exceed 40%. Interventions will likely be needed to maintain patency.

AVG may be a better choice for patients with a life expectancy of around 12–24 months who need permanent access. For those with a life expectancy under 12 months or poor surgical candidacy, a tunneled catheter as definitive access makes sense. When AVF is pursued in older individuals, proximal fistulas such as brachiocephalic have lower failure rates than distal radiocephalic fistulas.

Access plans should be reassessed regularly as clinical status changes. Patients who start hemodialysis with a catheter should convert to a fistula if life expectancy allows time for maturation, but otherwise a graft or persistent catheter may be more appropriate based on surgical risk [77]. The aim is not to abandon AVF completely. It is about matching access type to individual circumstances. For some frail older individuals with limited life expectancy, simpler access options may better align with their clinical circumstances and goals of care (Table 4).

## 6. Hemodialysis Membrane Selection in Older Adults

The choice of hemodialysis membranes in older adults should reflect overall clinical vulnerability rather than clearance targets alone. Advanced age is frequently associated with cardiovascular disease, impaired autonomic regulation, malnutrition, and limited vascular access. These factors strongly influence dialysis tolerance. In practice, the trade-off often involves the risk of intradialytic hypotension, albumin balance, and the feasibility of the prescribed blood flow and treatment time.

### 6.1. Biocompatibility and Membrane Materials

Synthetic polymer membranes, most commonly polysulfone (PSf) and polyethersulfone (PES), have largely replaced early unmodified cellulose dialyzers. This shift reflects improvements in biocompatibility, chemical resistance, and production consistency rather than a single dominant mechanism. Reduced complement activation is achieved through surface modification and increased hydrophilicity [78]. Protein adsorption and downstream leukocyte and platelet activation are also shaped by surface chemistry [79,80]. While masking of hydroxyl groups is a key mechanism underlying the improved compatibility of modified cellulose compared with regenerated cellulose, appropriately engineered modified cellulosic membranes can approach the biocompatibility of synthetic membranes. However, synthetic polymers generally maintain advantages in overall hemocompatibility [78,79,80,81]. These observations indicate that surface treatment and polymer formulation are more relevant than base material alone [79,80].

### 6.2. Permeability and High-Flux Membranes

Membrane permeability is particularly relevant in older individuals with limited cardiac reserve and a high burden of cardiovascular comorbidity. In this setting, higher water permeability and a broader sieving profile may increase the removal of middle molecules, but the prescription must remain aligned with hemodynamic tolerance [79]. Observational evidence suggests that high-flux hemodialysis may be associated with improved cardiovascular outcomes in selected older populations. In a retrospective cohort of 359 older individuals on maintenance hemodialysis, high-flux membranes were associated with lower cardiovascular mortality compared with low-flux dialysis. In contrast, all-cause mortality was not reduced [82]. Similar signals were reported in subgroup analyses of the Membrane Permeability Outcome study, in which patients with lower baseline serum albumin appeared to benefit from high-flux treatment, especially those with diabetes mellitus; these findings were subgroup-specific and should be interpreted accordingly [83]. No overall survival advantage was observed in the randomized population, supporting a selective rather than universal approach.

Low-flux membranes retain a role in a small subset of frail patients whose intradialytic hypotension persists despite extended treatment time, cool dialysate, and rigorous dry weight optimization. Their lower hydraulic permeability curtails unintended convective losses and reduces session-to-session osmotic variation, which may translate to better haemodynamic stability when cardiovascular reserve is severely reduced. No randomized data support this use; clinical experience suggests it as a holding measure while the causes of haemodynamic instability are addressed, not as a long-term prescription [37].

According to a recent European consensus statement, convective therapies such as hemodiafiltration, which combine diffusive and convective solute transport mechanisms, may be associated with improved intradialytic hemodynamic tolerance compared with conventional high-flux hemodialysis. This potential benefit appears particularly relevant in older individuals and in those with cardiovascular comorbidity, based on observational evidence and physiological considerations [23].

### 6.3. Heterogeneity Within Polysulfone Membranes

Despite being grouped under a single material category, PSf membranes differ substantially in biocompatibility. Experimental and comparative studies of commercially available PSf dialyzers have demonstrated marked variability in platelet adhesion, neutrophil activation, and reactive oxygen species production [84,85]. The amount of fibrinogen adsorbed at the membrane surface appears to be a key determinant of these effects, with higher fibrinogen adsorption correlating with increased GPIIb/IIIa-mediated platelet activation and Mac-1 mediated neutrophil activation [84]. PSf membranes incorporating hydrophilic polymer modifications induce lower cellular activation and oxidative stress than conventional formulations [84], and clinical studies have confirmed improvements in platelet-derived microparticle levels and endothelial function with modified membranes [86], reinforcing the clinical relevance of surface engineering in blood–membrane interactions.

### 6.4. Medium Cut-Off Membranes: Access, Albumin, and Outcomes

Vascular access limitations are common in advanced age, owing to higher rates of arteriovenous fistula non-maturation and prolonged catheter use [87]. In this setting, medium cut-off (MCO) membranes have been introduced as an alternative to conventional high-flux dialyzers. These membranes, typically composed of polyarylethersulfone blended with polyvinylpyrrolidone, enable enhanced removal of large middle molecular weight solutes, including those up to approximately 45 kDa, while keeping albumin leakage within a controlled range compared with high-flux membranes. However, losses are measurable [88,89,90]. Compared with standard high-flux membranes, MCO dialyzers achieve higher reduction ratios for free light chains and other large middle molecules [89].

Clinical outcome data are still evolving. In addition to observational analyses, a systematic review and meta-analysis including randomized and nonrandomized studies comparing MCO and high-flux membranes reported improvements in several patient-important outcomes (including quality of life, pruritus, recovery time, and restless legs syndrome), with little to no difference in all-cause mortality and serious adverse events; reductions in hospitalization and infection rates were also observed, although certainty varied across endpoints and larger definitive trials are still needed [91]. In an observational cohort study using weighted analysis, MCO membranes were associated with fewer hospitalizations and fewer nonfatal cardiovascular events compared with high-flux hemodialysis; residual confounding cannot be excluded and causality cannot be inferred [92]. A prospective observational study further showed that MCO membranes maintained superior clearance of large middle molecules at blood flow rates around 250 mL/min, outperforming both conventional hemodialysis and online hemodiafiltration specifically for large middle molecular weight solutes [93].

This may matter when catheter flow limits effective delivered Qb or when higher extracorporeal flows are poorly tolerated. In these situations, the enhanced permeability characteristics of MCO membranes may partially compensate for lower achievable blood flow rates. Albumin loss requires attention in frail or malnourished individuals. Studies consistently report higher intradialytic albumin losses with MCO membranes, although these are generally modest and may stabilize over time, with variable effects on serum albumin levels [89,90]. These findings should be interpreted with caution, as much of the available evidence derives from observational studies or registry analyses. Consequently, the reported associations should not be interpreted as demonstrating a causal relationship.

### 6.5. Tolerance, Inflammation, and Adjunctive Membrane Strategies

Chronic inflammation is a common characteristic of CKD [94]. In older dialysis patients it reflects the combined effects of uremia, immunosenescence, and multimorbidity. In a randomized controlled trial, treatment with MCO membranes was associated with reductions in circulating inflammatory mediators, including soluble tumor necrosis factor receptor 1 and free light chains; the trial was biomarker-driven and not powered for clinical endpoints [95].

Hemodynamic instability during dialysis represents a significant source of morbidity in older adults. Although high-flux and MCO membranes are sometimes perceived as increasing the risk of intradialytic hypotension, this complication is more closely related to ultrafiltration rate, impaired vascular refilling, and underlying cardiac or autonomic dysfunction than to membrane permeability itself [96,97]. Rapid fluid removal has been associated with myocardial injury and adverse cardiovascular outcomes; repetitive ischemic myocardial injury (“stunning”) is a plausible downstream pathway [45,47]. Dialysis intensity should also be adjusted cautiously. Excessively rapid solute removal may predispose susceptible patients to dialysis disequilibrium symptoms, particularly during treatment initiation, re-initiation, or after prolonged underdialysis [98].

Recognition of the role of large middle molecules and protein-bound uremic toxins in cardiovascular disease has driven the development of membranes with enhanced selectivity. Although MCO membranes improve middle-molecule clearance, their effect on protein-bound solutes remains limited because high protein binding reduces the free fraction available for diffusion [99,100]. Advances in polymer blending and surface modification have nevertheless improved selectivity while preserving essential plasma proteins [101,102,103].

Additional approaches target oxidative stress and inflammation. Membranes incorporating surface-bound antioxidants, most commonly vitamin E derivatives, aim to reduce oxidative stress at the blood–membrane interface, with permeability within the same membrane class being broadly comparable [104,105]. Clinical studies report reductions in lipid peroxidation and improvements in erythrocyte stability and erythropoietin responsiveness, although evidence for hard clinical outcomes is lacking [106,107]. Adsorptive membranes based on polymethylmethacrylate-type polymers represent another adjunctive strategy. These membranes combine diffusive clearance with protein adsorption through their symmetric porous structure, enabling removal of inflammatory mediators and selected middle-molecular-weight solutes that escape conventional dialysis [108]. Comprehensive proteomic analysis has documented efficient adsorption of multiple cytokines, including IL-6, IL-1β, and TNF-α, as well as protein-bound uremic toxins such as p-cresyl sulfate and indoxyl sulfate, although the in vivo relevance of this adsorption for protein-bound toxins remains uncertain [109,110]. The adsorptive capacity shows temporal dependency and reaches saturation within hours of membrane-blood contact, limiting its therapeutic window [111].

Clinical investigations have documented reductions in circulating inflammatory markers. Prospective trials measuring IL-6, IL-8, and C-reactive protein concentrations demonstrated statistically significant decreases after three months of PMMA membrane use compared with polysulfone controls [112]. Symptomatic benefits associated with polymethylmethacrylate (PMMA) membranes have been reported, particularly regarding dialysis-related pruritus. Small observational studies and crossover investigations have suggested a reduction in uremic pruritus severity following the use of PMMA membranes, potentially mediated by the adsorptive removal of pruritogenic middle-molecular-weight solutes; however, the underlying mechanisms remain incompletely elucidated [113].

Evidence regarding survival outcomes derives primarily from observational data. Analysis of the Japanese nationwide dialysis registry demonstrated lower adjusted hazard ratios for mortality among patients treated with PMMA membranes compared with polysulfone dialyzers, after adjustment for demographic, nutritional, and inflammatory variables. Nonetheless, no randomized controlled trials have confirmed a survival benefit, and residual confounding cannot be excluded in registry-based analyses [114].

### 6.6. Asymmetric Cellulose Triacetate Membranes

Asymmetric cellulose triacetate (ATA) membranes represent an evolution from earlier symmetric cellulosic designs. In the SOLFA multicenter randomized crossover study, ATA allowed substantially reduced anticoagulation (approximately half the usual heparin dose on average in the final session) and a higher proportion of sessions completed with reduced or zero heparin, with lower dialyzer fiber clotting compared with commonly used high-permeability synthetic dialyzers [115]. In post-dilution online hemodiafiltration, studies report adequate removal of small- and mid-sized solutes with generally limited albumin leakage, although performance depends on prescription parameters [116]. Across pre- and post-dilution modalities and varying substitution volumes, albumin leakage is membrane- and prescription-dependent, particularly at high convective volumes [117]. Proteomic analyses show a distinct adsorption profile and, in comparative studies, lower protein adsorption than conventional symmetric CTA [118]. ATA may be considered in patients with suspected hypersensitivity to synthetic membranes. Clinical outcome data remain limited, and no randomized trials have compared survival between ATA and synthetic high-flux membranes in older populations (Table 5).

## 7. Conservative Kidney Management

### 7.1. Defining Conservative Care

Conservative kidney management (CKM) is a comprehensive non-dialytic treatment strategy for end-stage kidney disease that represents a viable alternative to dialysis in older, frail individuals with substantial comorbidity [119,120]. CKM is not withdrawal of care but rather active disease management. It encompasses holistic treatment of advanced CKD including medical interventions to slow progression when acceptable to patients, symptom-directed therapies, management of electrolyte and bone-mineral disorders, anemia treatment, dietary modification, advanced care planning, and palliative support when appropriate [121].

CKM prioritizes quality of life over quantity. Dialysis prolongs survival in most patients, but it may not align with the goals and values of all older individuals with multiple comorbidities and functional impairment.

### 7.2. Survival Outcomes

Multiple systematic reviews have compared survival between CKM and dialysis. Dialysis generally prolongs survival compared with CKM in the overall end-stage kidney disease population [119,122]. This survival benefit diminishes or disappears in specific subgroups though: patients aged ≥80 years, patients with high comorbidity burden, and patients with significant frailty [123,124].

Buur et al. conducted a systematic review identifying 25 studies comparing CKM with dialysis [125]. Overall, CKM was associated with shorter survival than dialysis. Several studies showed similar mortality rates in patients ≥ 80 years old or older individuals with multiple comorbidities. A 2023 review found that among the oldest and frailest individuals with the most comorbidities, survival was similar across modalities [119].

Advanced age, frailty, and comorbid conditions carry substantial baseline mortality risk. When baseline risk is high, initiating dialysis may add relatively little survival benefit while imposing considerable treatment burden [126]. Optimal care requires reconciling clinical objectives with patient-reported outcomes through open dialog to determine the most suitable treatment strategy.

### 7.3. Quality of Life and Symptom Burden

Survival is only one outcome dimension. Quality of life, symptom burden, hospitalization, and place of death are equally important for patients prioritizing comfort over longevity.

Evidence on quality of life shows mixed results but generally suggests CKM may offer advantages in certain domains. A systematic review found similar quality of life among older individuals with end-stage kidney disease on CKM versus dialysis [127]. Some studies reported better physical quality of life in CKM patients, possibly because they avoided dialysis-related complications and had less advanced disease at baseline [128].

Multiple studies have found similar prevalence and severity of symptoms such as fatigue, pruritus, and sleep disturbance among CKM and dialysis patients [129,130]. Some studies reported slightly higher symptom scores in dialysis patients, which may reflect dialysis-related complications [131]. CKM patients reported higher kidney disease burden but not necessarily worse overall quality of life [132].

Hospitalization rates favor CKM. A value-based comparison found than CKM patients had 352.7 hospital-free days per year compared with 282.7 for dialysis patients [133]. CKM patients also more frequently die at home or in hospice, whereas dialysis patients more often die in hospital, sometimes receiving aggressive interventions near the end of life [134]. For patients who value dying at home surrounded by family, CKM facilitates this preference.

### 7.4. Patient and Clinician Perspectives

Understanding CKM selection requires examining decision-making processes. Sakthiel et al. conducted a systematic review identifying factors that influence CKM choice [135]. Patients valued maintaining their current lifestyle, avoiding treatment burden, and prioritizing quality of life over quantity. Life expectancy information influenced decisions. Patients were more likely to choose CKM when informed that dialysis might not extend their survival.

Clinicians have historically viewed dialysis as the active treatment modality and CKM as failure of medicine [136]. Attitudes are evolving, however, particularly among recently qualified nephrologists who recognize that for carefully selected patients CKM represents appropriate care. Research showing similar life expectancy for older individuals with dementia or ischemic heart disease regardless of treatment modality has made nephrologists more comfortable recommending CKM [137].

Barriers to CKM implementation include limited clinical guidance, time constraints for sensitive discussions, uncertainty about prognosis, and concerns about appearing nihilistic [138]. Developing CKM pathways requires institutional support, multidisciplinary collaboration, and clinician education about appropriate patient selection and symptom management strategies. In practical terms, multidisciplinary management may include structured geriatric assessment at dialysis initiation, periodic reassessment of functional status, coordinated medication review, and integration of palliative care expertise when symptom burden becomes prominent. Such collaborative models may help translate geriatric principles into routine nephrology practice.

### 7.5. Clinical Implementation

Practical CKM delivery addresses multiple aspects of patient care. Appropriate candidates include patients aged ≥80 years with multiple comorbidities, patients with very high frailty scores or severe functional impairment, patients with dementia or terminal illness, and patients who prioritize quality over quantity of life after informed discussion [139].

Clinical management protocols cover several domains. Dietary modification with protein restriction if tolerated, volume management through salt restriction and diuretics for those with residual function, symptom control using antiemetics, antipruritic agents, and pain management as needed. Anemia is managed with erythropoiesis-stimulating agents and iron supplementation. Bone-mineral disorders and cardiovascular risk factors require ongoing attention, along with psychosocial support [140,141].

Advance care planning is essential and should include discussions about goals of care, do-not-resuscitate preferences, hospitalization preferences, and end-of-life care plans [141]. These conversations need to start early and continue as clinical status changes.

While less intensive than dialysis, CKM requires regular follow-up. Monthly visits are suggested for patients with eGFR < 15 mL/min/1.73 m^2^, with more frequent monitoring during acute changes [142]. Laboratory monitoring includes metabolic panels, hemoglobin, parathyroid hormone, and nutritional markers.

Transitioning to hospice care becomes necessary when death is approaching to ensure appropriate symptom management and support. Many patients on CKM ultimately require hospice services. Median survival after hospice enrollment ranges from 1–3 months in various studies [143].

### 7.6. Shared Decision-Making Framework

The decision between dialysis and CKM should emerge from structured shared decision-making that incorporates patient values, prognostic information, and realistic expectations. Clinicians need to provide realistic survival estimates for both dialysis and CKM based on age, comorbidities, and frailty status. Treatment burden also requires thorough explanation, including time commitment, symptoms, complications, and functional impacts of each approach.

Values clarification is critical. Patients should have opportunities to explore their priorities around longevity versus quality of life, independence, family time, and avoiding hospitalizations. Decision aids can help, along with involving family members and allowing time for reflection. Initial decisions can be revisited if circumstances or preferences change.

Recent guidelines from multiple professional societies emphasize this shared decision-making framework and move away from default dialysis initiation toward individualized assessment of whether dialysis aligns with patient goals [144,145].

Interpretation of these findings requires caution because comparisons between conservative kidney management and dialysis are particularly vulnerable to selection bias. Patients who choose conservative care often differ systematically from those initiating dialysis in terms of comorbidity burden, functional status, and personal goals of care. In addition, differences in the integration of palliative and supportive care between treatment groups may influence reported quality-of-life outcomes.

## 8. Quality of Life and Patient-Centered Outcomes

### 8.1. Beyond Survival

Traditional nephrology outcome research has focused heavily on mortality and laboratory parameters like serum phosphorus, Kt/V, and hemoglobin. These metrics are treated as primary endpoints. They only capture fragments of what constitutes meaningful care for frail older individuals though. Patients prioritize different outcomes: symptom relief, functional independence, time with family, avoiding hospitalizations, and maintaining dignity [146].

This creates potential for misaligned care. A nephrologist might view well-controlled laboratory values as success while the patient feels miserable from dialysis side effects and treatment burden. Slightly elevated phosphorus might be acceptable to patients if dietary flexibility improves their quality of life.

### 8.2. Recovery Time After Dialysis

Post-dialysis recovery time profoundly impacts quality of life but receives insufficient attention. Studies using patient-reported outcomes found that 47–73% of hemodialysis patients require more than 2 h to recover after treatment, and 20% need more than 12 h [36,147]. This prolonged fatigue limits the ability to work, socialize, or perform daily activities. It consumes not just dialysis treatment time but substantial additional hours each week.

Factors associated with longer recovery include higher ultrafiltration rates, intradialytic hypotension, female gender, lower serum albumin, heart failure, and higher body weight [148]. UFR ≥ 13 mL/kg/hour is linked to significantly longer recovery time in adjusted analyses [147]. Aggressive dialysis may achieve better solute clearance, but the trade-off is often prolonged post-dialysis debility that compromises quality of life.

For frail older individuals, extended recovery time has major consequences. A patient needing 6–8 h to recover from each dialysis session loses 18–24 h weekly to treatment-related incapacity, nearly 15% of their total time. These considerations should factor into discussions about modifying dialysis prescriptions.

### 8.3. Functional Decline

Functional status often deteriorates after dialysis initiation in older individuals, which contradicts the expectation that correcting uremia improves function. Kurella Tamura et al. evaluated changes in functional status among 3702 older individuals adults initiating dialysis and found marked functional decline from 3 months before to 3 months after initiation [149]. Many patients who were previously functional became unable to walk or perform activities of daily living, and only a minority returned to baseline function.

Goto et al. examined associations between dialysis initiation and functional status in older adults [150]. Functional decline occurred in 57% of patients in the first 6 months after starting dialysis. Older age, female gender, greater comorbidity burden, and lower baseline functional status all predicted decline, which are exactly the characteristics that define frail patients.

Multiple factors explain this functional deterioration. Hemodynamic stress from dialysis, vascular access complications, hospitalizations, and a time burden that limits physical activity all play a role. Nutritional management also represents an important component of care in frail dialysis patients. Older individuals undergoing hemodialysis are particularly vulnerable to protein–energy wasting (PEW), which is strongly associated with reduced functional status, increased hospitalization, and mortality. Early nutritional assessment and targeted dietary interventions may therefore contribute to preserving physical function and improving patient-centered outcomes in this population [52,100]. Patient selection may also matter since those starting dialysis may be frailer than those choosing conservative management. Regardless of the mechanisms involved, dialysis does not reliably restore or preserve function in older individuals. Assumptions that guide dialysis initiation decisions need to account for this reality.

### 8.4. Integrating Patient-Centered Outcomes

Measuring what matters to patients requires different tools than traditional clinical metrics. Patient-reported outcome measures (PROMs) capture symptoms, function, and quality of life directly from patients rather than inferring these from laboratory values [151]. Validated instruments for hemodialysis populations include the Kidney Disease Quality of Life (KDQOL) instrument, which assesses physical function, pain, general health, emotional well-being, social function, and kidney disease-specific concerns [152].

Routine integration of PROMs into clinical care can identify problems that are invisible in traditional assessment like uncontrolled symptoms, functional limitations, depression, and caregiver stress. This information can then inform treatment adjustments, symptom management interventions, and goals-of-care discussions.

For frail older individuals, relevant outcomes extend beyond standard measures of quality of life. Time spent with family matters. So does the ability to attend meaningful events, independence in self-care, and avoiding nursing home placement. Many patients want to die at home if possible, avoid futile interventions, and maintain dignity throughout their illness.

## 9. Future Directions

### 9.1. Research Gaps

Substantial evidence gaps persist in this area. Randomized controlled trials specifically examining personalized dialysis approaches in frail older populations remain scarce. The trials that informed current guidelines predominantly enrolled younger, healthier patients, which limits how well we can apply them to frail older patient cohorts.

Several research priorities emerge. Randomized trials comparing incremental and standard hemodialysis in patients with residual kidney function would be valuable, measuring both clinical and quality-of-life outcomes. Comparative effectiveness studies are needed for vascular access strategies based on frailty status and life expectancy. UFR-reduction interventions tested specifically in frail patients could help guide practice. Prospective studies comparing CKM with dialysis in well-matched older patient cohorts could address selection bias through careful design. Patterns and outcomes of dialysis discontinuation among older individuals who choose to stop also warrant investigation.

### 9.2. Implementation Science

Successfully implementing personalized approaches requires addressing practical barriers. Dialysis facilities face financial pressures that incentivize standardized care and maximization of throughput. Dialysis companies may resist incremental hemodialysis despite patient benefits because twice-weekly schedules generate lower revenue than thrice-weekly schedules.

Payment models need to reward quality and patient-centered care rather than session quantity. Clinical decision support tools could facilitate frailty assessment and incorporate results into treatment planning. Staff education about geriatric principles, frailty assessment, and individualized care approaches would help. Quality measures should reflect patient-centered outcomes rather than just biochemical targets. Clinical pathways and protocols that support personalized dialysis prescriptions also need to be developed and disseminated across facilities.

### 9.3. Training and Education

Current nephrology training emphasizes technical aspects like vascular access procedures, dialysis prescription calculations, and biochemical management. Exposure to geriatric medicine is limited. Training programs should integrate comprehensive geriatric assessment, frailty evaluation, advance care planning, and shared decision-making skills into the curriculum. Fellowship programs could offer geriatric nephrology rotations or incorporate geriatrics faculty into teaching rounds.

Practicing nephrologists need continuing education in these competencies to adapt to the aging dialysis population. Professional societies can facilitate this through dedicated conference sessions, online educational modules, and practice guidelines that emphasize individualized approaches for older individuals.

### 9.4. Integrating Geriatric Principles

Optimal care for frail older dialysis patients requires integrating nephrology expertise with geriatric medicine principles. This integration might occur through collaborative care models where geriatricians co-manage complex older dialysis patients by performing comprehensive geriatric assessments and providing input on goals of care, medication management, and functional optimization.

Alternative models include training nephrologists in geriatric competencies or developing specialized geriatric nephrology clinics staffed by clinicians with dual expertise. The key is ensuring that geriatric considerations like frailty, cognitive function, fall risk, polypharmacy, and multimorbidity inform nephrology care plans regardless of the specific organizational structure chosen (Table 6).

Future research should prioritize randomized controlled trials specifically designed for frail and older dialysis populations, as the current evidence base remains largely derived from observational analyses or subgroup evaluations of trials conducted in younger cohorts.

## 10. Conclusions

The dialysis population has undergone a demographic transformation, becoming older, frailer, and more comorbid. Treatment approaches need to evolve accordingly. Standard hemodialysis protocols derived from younger populations may impose substantial burdens on frail older individuals without proportional benefits.

Frailty assessment provides crucial prognostic information and should be routinely integrated into nephrology practice. Validated tools exist that are feasible for clinical implementation and predict outcomes independent of traditional risk factors. Identifying frailty enables better risk stratification and helps inform treatment planning.

Dialysis prescription flexibility allows reduction in treatment burden while maintaining adequate solute clearance. Individualization should start with modality selection, as tolerability and hemodynamic stability are crucial in frail older individuals. Hemodiafiltration may provide better intradialytic tolerance and middle-molecule removal in suitable candidates. At the same time, HDx and acetate-free biofiltration represent valuable options when achieving high convection volumes is difficult or intradialytic hypotension risk is high. Incremental hemodialysis remains appropriate for patients with residual kidney function; in selected frail individuals, twice-weekly treatment can be a reasonable starting point, with intensity increased as residual function declines or symptoms emerge.

However, the enhanced permeability of medium cut-off membranes may also result in measurable albumin losses during dialysis sessions. This aspect is particularly relevant in older individuals who frequently present with hypoalbuminemia or protein-energy wasting and should therefore be considered when selecting dialysis membranes.

Ultrafiltration strategies should prioritize avoiding intradialytic hypotension and prolonged recovery time rather than achieving aggressive dry weight targets at all costs. Slower UFR through longer sessions or increased frequency improves tolerability when combined with a realistic assessment of volume status, and this does not necessarily compromise clinical outcomes.

Vascular access decisions need to incorporate frailty assessment, life expectancy estimation, and patient preferences. Reflexively pursuing fistula-first for all patients does not make sense. For frail older individuals with limited survival, AVG or definitive catheter use may be better choices than AVF creation given the prolonged maturation time and uncertain success.

Conservative kidney management represents a viable alternative to dialysis for carefully selected patients, particularly those aged ≥80 years with multiple comorbidities, high frailty, or preferences that prioritize quality over quantity of life. Dialysis generally prolongs survival, but this benefit diminishes in frail older populations. CKM may offer quality-of-life advantages by reducing treatment burden and hospitalizations.

Reimbursement structures in many healthcare systems may also influence dialysis prescription patterns, often favoring standardized thrice-weekly schedules over more individualized treatment approaches. Shared decision-making should guide treatment planning across all these considerations. Patient values, preferences, and realistic prognostic information need to be incorporated into decisions. Optimal care for frail older individuals requires flexible approaches tailored to individual circumstances rather than rigid adherence to population-level protocols.

Developing expertise in personalized approaches has become essential for nephrologists and dialysis teams as the dialysis population continues to age. Success requires clinical knowledge and systems change, including payment models that support individualized care, clinical decision support tools, enhanced training in geriatric principles, and cultural shifts that value patient-centered outcomes alongside traditional metrics.

The strategies discussed in this review should therefore be interpreted as pragmatic approaches supported mainly by observational evidence and clinical experience, while awaiting confirmation from prospective randomized studies specifically designed for frail dialysis populations.

## Figures and Tables

**Table 1:** Frailty Assessment Tools in Hemodialysis Populations.

Tool	Domains Assessed	Advantages/Limitations	References
Fried Frailty Phenotype	Five components: unintentional weight loss, self-reported exhaustion, low physical activity, weak grip strength, slow walking speed. ≥3 criteria = frail, 1–2 = pre-frail, 0 = robust.	Extensively validated in dialysis populations. Requires functional capacity for performance-based testing, limiting use in very debilitated patients.	[12]
Clinical Frailty Scale (CFS)	Nine-point scale from very fit (1) to terminally ill (9) based on clinical judgment.	Simple, requires no equipment or patient cooperation. Appropriate for routine clinical use. Correlates with adverse outcomes in dialysis patients.	[4,13]
FRAIL Scale	Self-reported questionnaire: Fatigue, Resistance, Ambulation, Illnesses, Loss of weight.	Brief and simple, facilitating implementation. May underestimate frailty severity compared to performance measures.	[14]
Short Physical Performance Battery (SPPB)	Objective assessment of balance, gait speed, and chair stand performance.	Strong prognostic value. Requires patients capable of performing physical tasks, limiting applicability in severely frail individuals.	[15]
Comparative Studies	Different scales yield varying prevalence estimates but most correlate with increased mortality, hospitalization, and cardiovascular events.	Choice should balance practical constraints, clinical context, and precision. All tools demonstrate independent prognostic value beyond age and comorbidity.	[16]

Table 1 Validated frailty assessment instruments for hemodialysis populations with specific characteristics, clinical applications, and reference citations. Abbreviations: CFS, Clinical Frailty Scale; FRAIL, Fatigue–Resistance–Ambulation–Illnesses–Loss of weight; SPPB, Short Physical Performance Battery.

**Table 2:** Dialysis Frequency and Duration Approaches.

Modality	Schedule	Advantages in Frail Older Individuals	References
Standard Hemodialysis	3×/week, 3.5–4 h/session	Established protocols, adequate for anuric patients. Imposes substantial burden: 12 h weekly in unit plus travel, recovery time affects 47–73% patients for >2 h.	[36,38]
Incremental Hemodialysis	Twice-weekly initially, transition to 3×/week as residual function declines	Lower treatment burden, preserves residual kidney function, reduced vascular access trauma, decreased intradialytic complications. Particularly relevant for patients with residual function and higher treatment burden.	[39,40,41]
Once-weekly Hemodialysis	1×/week (investigational)	Feasible in patients with significant residual function when combined with aggressive dietary management and close monitoring. Still primarily research setting.	[42]
Extended Hours Options	Nocturnal (6–8 h, 3–6×/week) or short daily (2–3 h, 5–6×/week)	Gentler ultrafiltration, better hemodynamic stability for nocturnal. Limited applicability in frail populations due to prolonged immobility concerns, increased access demands, and time burden.	[43,44]

Table 2 Alternative hemodialysis schedules with specific advantages and limitations for frail older individuals, emphasizing individualization based on residual kidney function and patient tolerance. Abbreviations: h, hours.

**Table 3:** Strategies to Reduce Ultrafiltration Rate and Prevent Intradialytic Hypotension.

Strategy	Implementation Details	References
Extended Treatment Time	Increase session duration from 3.5 to 4.5–5 h. Proportionally reduces UFR, improves hemodynamic stability. DOPPS demonstrated associations between longer treatment time and reduced mortality.	[57]
Increased Frequency	Four times weekly schedule distributes fluid removal across additional sessions, reducing per-session UFR. Benefits patients with large interdialytic weight gains who cannot extend individual sessions.	[64]
Dry Weight Optimization	Rigorous assessment using clinical criteria (blood pressure patterns, edema, dyspnea, post-dialysis fatigue) combined with bioimpedance analysis. Regular reassessment prevents pursuing unrealistic targets.	[58]
Dietary Management	Sodium restriction and interdialytic fluid intake education reduce weight gain between sessions, permitting lower UFR. Loop diuretics may assist volume control in patients with residual function.	[64]
Cool Dialysate	Temperature reduction to 35.5 °C versus 37 °C reduces intradialytic hypotension incidence by minimizing vasodilation.	[59]
Dialysate Profiling	Adjusting sodium and bicarbonate composition during session may improve hemodynamic stability, though evidence remains mixed.	[60]
Blood Volume Monitoring	Hematocrit or relative blood volume measurement systems trigger UFR adjustments, reducing hypotension risk.	[65]

Table 3 Evidence-based interventions to reduce ultrafiltration-related complications in frail hemodialysis patients, emphasizing hemodynamic stability and quality of life preservation. Abbreviations: DOPPS, Dialysis Outcomes and Practice Patterns Study; UFR, ultrafiltration rate.

**Table 4:** Vascular Access Options in Frail Older Individuals: Comparative Considerations.

Access Type	Characteristics in Older Patients	Considerations for Frail Patients	References
Arteriovenous Fistula (AVF)	Maturation time: 2–6 months. Primary failure rates: 20–60% overall, up to 42.1% for radiocephalic in octogenarians vs. 5.6% for brachiocephalic. Superior long-term patency when successful.	Prolonged catheter dependence during maturation. High failure rates in older individuals vascular disease. May not benefit patients with life expectancy <12–18 months. Proximal AVF preferred over distal when pursued.	[69,70,71]
Arteriovenous Graft (AVG)	Shorter maturation (2–4 weeks). Lower primary failure than AVF in older individuals. Higher intervention rates than AVF but less catheter dependence time.	Appropriate for life expectancy 12–24 months requiring permanent access. Reduced maturation time versus AVF. Suitable option for patients with poor vessels or high AVF failure risk.	[72,73]
Central Venous Catheter (CVC)	Immediate usability. Higher infection risk. Lower patency than permanent access. Associated with increased mortality when compared to AVF/AVG long-term.	Consider as definitive access for life expectancy <12 months or poor surgical candidacy. Avoids procedures unlikely to yield benefit. Spares very frail patients from surgical risk.	[77]
Frailty-Guided Selection	Frail and very frail patients show higher AVF non-maturation rates, shorter survival, higher mortality within 3 years of access creation.	2019 KDOQI update advocates life-plan approach with shared decision-making incorporating life expectancy, frailty status, vascular anatomy, and patient preferences rather than universal fistula-first.	[74,76]

Table 4 Vascular access options for frail older individuals hemodialysis patients with comparative analysis of maturation times, failure rates, and suitability based on life expectancy and frailty status. Abbreviations: AVF, arteriovenous fistula; AVG, arteriovenous graft; CVC, central venous catheter; KDOQI, Kidney Disease Outcomes Quality Initiative.

**Table 5:** Overview of the application of filters in personalized dialysis prescription.

Membrane Type	Materials	Advantages in Older Adults	Limitations	Indications	References
High-Flux Synthetic	Polysulfone (PSf) Polyethersulfone (PES) Hydrophilic modified variants	High biocompatibility with reduced complement activation Effective middle molecule removal ↓ all-cause mortality in older individuals with low albumin (MPO subanalysis) HDF: improved hemodynamic tolerance	Heterogeneity among commercial PSf No proven benefit on all-cause mortality Requires prescription aligned with CV tolerance	Standard for older individuals with CV comorbidity, diabetes, low albumin	[23,78,79,80,81,83,84,85]
Medium Cut-Off (MCO)	Polyarylethersulfone + PVP Cutoff: up to ~45 kDa	↑ large middle molecule removal (FLC) even at low Qb (250 mL/min) Useful with limited vascular access (e.g., CVC) ↓ CV events and hospitalizations (observational) ↓ inflammatory mediators	↑ intradialytic albumin loss Limited clinical outcome data Caution in frail/malnourished patients	Older individuals with CVC, difficult AVF maturation, limited achievableQb	[88,89,90,92,93,95]
Vitamin E-coated	PSf/PES with immobilized α-tocopherol	↓ oxidative stress and lipid peroxidation Improved erythrocyte antioxidant status ↑ EPO response (meta-analysis)	Limited evidence on clinical outcomes (hard endpoints) Benefits mainly on biomarkers	Older individuals with high oxidative stress, EPO-resistant anemia	[104,105,106,107]
PMMA (Adsorptive)	Polymethylmethacrylate Symmetric porous structure	Cytokine adsorption (IL-6, IL-1β, TNF-α) ↓ inflammatory markers (CRP) Improvement in uremic pruritus ↓ mortality (Japanese registry)	Adsorptive capacity saturates within hours No RCT on survival Possible residual confounding	Older individuals with high inflammation, severe uremic pruritus	[108,109,110,111,112,113,114]
Asymmetric Cellulose Triacetate (ATA)	Asymmetric triacetate Modified cellulosic polymer Smooth inner surface	Lower thrombogenicity profile: allows safe heparin reduction to 50% High diffusive transport with minimal albumin loss (~1 g/session in HDF) Alternative for synthetic membrane hypersensitivity Lower protein adsorption vs. symmetric CTA	Limited long-term clinical outcome data No survival benefit studies vs. synthetic membranes Cost may be higher than standard PSf	Older individuals with bleeding risk requiring reduced anticoagulation, synthetic membrane allergy	[115,116,117,118]

Table 5 summarizes the main hemodialysis membrane types suitable for older adults, comparing materials, clinical advantages, limitations, and specific indications. The selection should reflect overall clinical vulnerability, vascular access status, and hemodynamic tolerance rather than clearance targets alone. Abbreviations: ATA = asymmetric cellulose triacetate; AVF = arteriovenous fistula; CRP = C-reactive protein; CTA = cellulose triacetate; CV = cardiovascular; CVC = central venous catheter; EPO = erythropoietin; FLC = free light chains; HDF = hemodiafiltration; IL = interleukin; MCO = medium cut-off; MPO = Membrane Permeability Outcome (study); PES = polyethersulfone; PMMA = polymethylmethacrylate; PSf = polysulfone; PVP = polyvinylpyrrolidone; Qb = blood flow; RCT = randomized controlled trial; TNF = tumor necrosis factor. The symbols ↓ and ↑ indicate decreases and increases, respectively.

**Table 6:** Practical Framework for Personalized Hemodialysis in Frail Older individuals.

Domain	Personalization Strategies	References
Initial Assessment	Integrate frailty screening into pre-dialysis evaluation using validated tools (CFS for rapid scoring, FRAIL scale < 5 min). Comprehensive geriatric evaluation including medication review, fall risk, cognitive screening when frailty identified.	[22]
Dialysis Prescription	Match intensity to individual circumstances: incremental HD (twice-weekly initially) for patients with residual function and minimal symptoms; standard thrice-weekly for anuric patients with modified session duration/UFR as needed. Consider residual renal function, interdialytic weight gain, functional status, symptom burden, patient preferences.	[39,40,42]
Ultrafiltration Management	Prioritize avoiding intradialytic hypotension over aggressive dry weight targets. Employ longer sessions, increased frequency, rigorous dry weight assessment with bioimpedance, sodium/fluid restriction education, cool dialysate (35.5 °C), blood volume monitoring. Accept modest volume excess when alternative is symptomatic hypotension and debilitating fatigue.	[57,58,59,65]
Vascular Access Selection	Life-plan approach: AVG for life expectancy 12–24 months requiring permanent access; tunneled catheter as definitive access for <12 months life expectancy or poor surgical candidacy; proximal AVF over distal when AVF pursued in older individuals. Incorporate frailty assessment, life expectancy estimation, patient preferences through shared decision-making.	[74,76,77]
Conservative Management Option	Appropriate candidates: ≥80 years with multiple comorbidities, very high frailty scores, severe functional impairment, dementia or terminal illness, patients prioritizing quality over quantity after informed discussion. Comprehensive non-dialytic management with symptom control, advance care planning, regular monitoring.	[119,139,140]
Shared Decision-Making	Structured process incorporating prognosis discussion with realistic survival estimates, treatment burden description, values clarification exploring patient priorities, decision support tools, family involvement, allowance for reassessment. Move from default dialysis initiation toward individualized assessment of goal alignment.	[9,144,145]

Table 6 Comprehensive clinical implementation framework for delivering personalized hemodialysis to frail older individuals across key decision domains, emphasizing individualized assessment and shared decision-making. Abbreviations: AVF, arteriovenous fistula; AVG, arteriovenous graft; CFS, Clinical Frailty Scale; FRAIL, Fatigue–Resistance–Ambulation–Illnesses–Loss of weight; HD, hemodialysis; UFR, ultrafiltration rate.

## Data Availability

No new data were created or analyzed in this study. Data sharing is not applicable to this article.
